# Are absorption and spontaneous or stimulated emission inverse processes? The answer is subtle!

**DOI:** 10.1007/s00340-019-7133-z

**Published:** 2019-01-21

**Authors:** Markus Pollnau

**Affiliations:** Department of Electrical and Electronic Engineering, Advanced Technology Institute, University of Surrey, Guildford, GU2 7XH UK

## Abstract

It is generally believed that absorption and stimulated emission are inverse processes, as both are driven by an external field, their strength is quantified by the same Einstein *B* coefficient, and they occur with a defined phase, opposite to each other, namely in phase and in anti-phase with the driving field, whereas spontaneous emission is a different process that occurs with an arbitrary phase with respect to a potential incident field. Recently, the phase relation in absorption and emission was shown to differ from this believe. Here it is verified via the amplitude–phase diagram and via the interference of sine waves that, precisely speaking, only the absorption process, in which a number *φ* + 1 of incident photons is decreased by one photon, and the emission process, in which a number *φ* of incident photons is increased by one photon, are truly inverse processes also in their phase. Particularly, this implies that absorption of a single incident photon and spontaneous emission of a photon into an empty mode are inverse processes in the amplitude–phase diagram.

## Introduction

In 1995, Nobel laureate Willis E. Lamb published a paper entitled “Anti-photon” in Applied Physics B [1], in which he expressed his dissatisfaction with how little a grip his scientific environment mastered upon the field of photonics. Particularly, he condemned the association of an electromagnetic wave with a particle—the photon. Besides many other examples, in one he reprimanded another Nobel laureate, Albert Einstein, for a fundamental conceptual mistake in his seminal paper from 1917 [2], when introducing a “new” (set within quotation marks by Lamb [1]) process of stimulated emission of radiation, quantified by the Einstein *B* coefficient. Lamb criticized Einstein for not having realized that, whereas the Einstein *A* coefficient of spontaneous emission cannot be a result of classical electromagnetic theory, “the classical Maxwell electrodynamics already made provision for both of the Einstein *B* coefficients of absorption and stimulated emission” [1]. Lamb then becomes explicit: “It would have made for much better physics if Einstein had recognized this fact, and had used his theory to calculate the value of the *A* coefficient for spontaneous emission in 1917, instead of leaving it to Dirac in 1927 to get the *A* coefficient from the quantum theory of radiation” [1]. Here Lamb referred to Ref. [3], albeit without citing it.

**Fig. 1:**
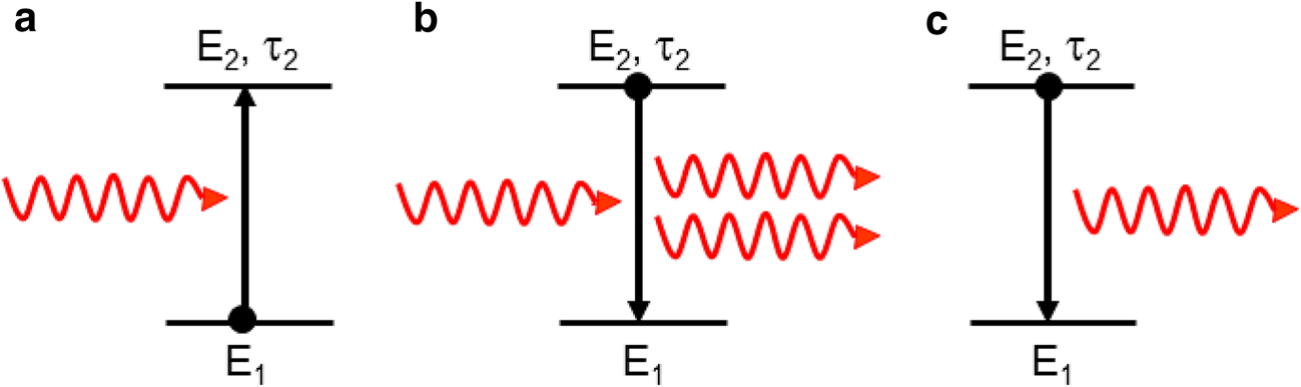
The processes of **a** absorption, **b** stimulated emission, and **c** spontaneous emission by a two-level atom with level energies *E*_1_ and *E*_2_ and an upper-state luminescence lifetime of *τ*_2_, as typically depicted in an optics text book

Despite Lamb’s dissatisfaction, I will use here the word photon, however, only in the sense of the quantized energy unit of light. In a 2nd semester university course on classical electrodynamics, we are typically instructed in the following way (Fig. 1):

- Spontaneous emission is a process, in which an atom in its excited state emits an electromagnetic field, comprising the energy of one photon, with an arbitrary phase with respect to a potentially existing incident field (but the students would learn later that spontaneous emission is actually driven by so-called vacuum fluctuations [4]).
- Stimulated emission (absorption) is a process, in which an incident electromagnetic field, comprising the energy of one photon, forces an atom in its excited (ground) state to oscillate and emit a second electromagnetic field, comprising the energy of one photon, that is in phase (anti-phase) with the incident field, such that these two fields interfere constructively (destructively) with each other and the energy is emitted (absorbed) by the atom.

To Lamb—and, for once, also to most of his scientific environment—it was crystal clear that absorption and stimulated emission are the two truly inverse processes. This seemed obvious, as (1) both processes are driven by a real incident electromagnetic field, (2) their strength is quantified by the same Einstein *B* coefficient, and (3) these two processes occur with a defined, opposite phase. In this paper, I will defend the position that the situation is, indeed, a bit more subtle—and that Einstein intuitively had a point.

## Absorption and emission in the amplitude–phase diagram

Recently, I emphasized [5] that, in stark contrast to the above-mentioned instructions, all quantitative semi-classical models suggest that, generally, emission of a photon at the resonance frequency of an atomic transition must occur with a phase that is 90° in lead of an incident field, whereas absorption must occur with a phase that lags 90° behind the resulting field. Only in this way energy is conserved. We understand this fact when dividing the emission or absorption process into its two parts, namely an oscillation of the electron cloud around the atomic core and the emission of an electromagnetic wave by this oscillating electron cloud. The Lorentz oscillator model, resulting in the complex susceptibility, or, equivalently, the mathematical Kramers–Kronig relations imply that the electron oscillation occurs at the mentioned phase difference of 90°. Whereas the near-field radiation of an oscillating electric dipole is rather complex [6, 7], with the phases of longitudinal and transverse components of the electric field changing with increasing distance from the dipole, the far-field radiation emitted by an atomic dipole is dominated by the transverse component and is in phase with the atomic dipole oscillation [6–8]. Therefore, the total phase difference is 90°.

**Fig. 2:**
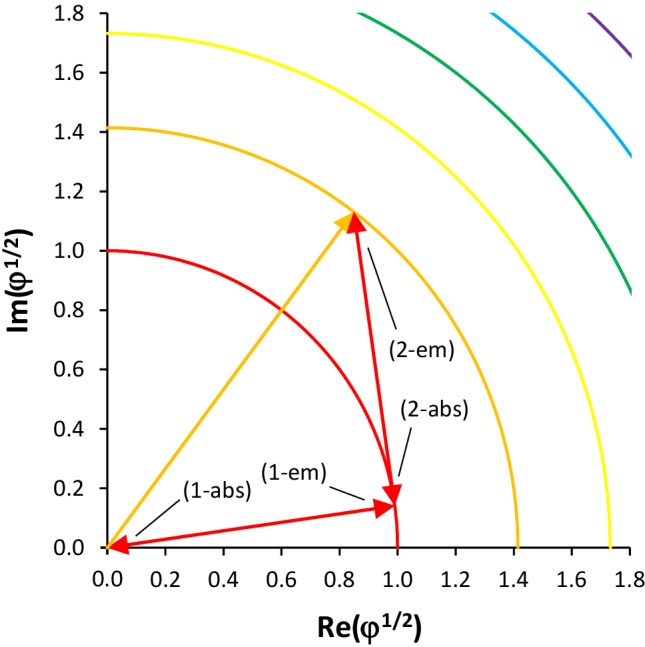
Quadrant of the amplitude–phase diagram. Incident and resulting fields, as well as absorption and emission processes are represented by electric-field vectors. Labels: a maximum of (1) or (2) photons is involved in an absorption (abs) or emission (em) process

Figure 2 shows an amplitude–phase diagram. The coordinate axes are in units of the electric-field amplitude, calibrated to the square root of the number of photons at the frequency *ν* that is resonant with the atomic transition. Consequently, the quarter circles denote the amplitudes of electric fields comprising 1 (red), 2 (orange), 3 (yellow), etc. photon energies *hν*. The *x*- and *y*-axes display the real and imaginary parts of the amplitude, respectively. The coordinate system rotates with e^*i*2*πνt*^. All red vectors denote electric fields comprising one photon, whereas the orange vector denotes an electric field comprising two photons. The angle between the two sets of red vectors is 90°. Four processes can be identified, including a maximum of either one (1) or two (2) photons and describing an absorption (abs) or emission (em) process. The four processes are (1-abs) absorption of the electric field of a single incident photon, (1-em) spontaneous emission of the electric field of a photon into an empty mode, (2-abs) absorption of the electric field of one out of two incident photons, and (2-em) stimulated emission of the electric field of a photon driven by a single incident photon. In each case, the initial electric field, the absorbed or emitted electric field (labelled), and the resulting electric field are shown. All four processes obey the law of energy conservation. The figure suggests that the processes (1-abs) and (1-em) are inverse and the processes (2-abs) and (2-em) are inverse.

**Fig. 3:**
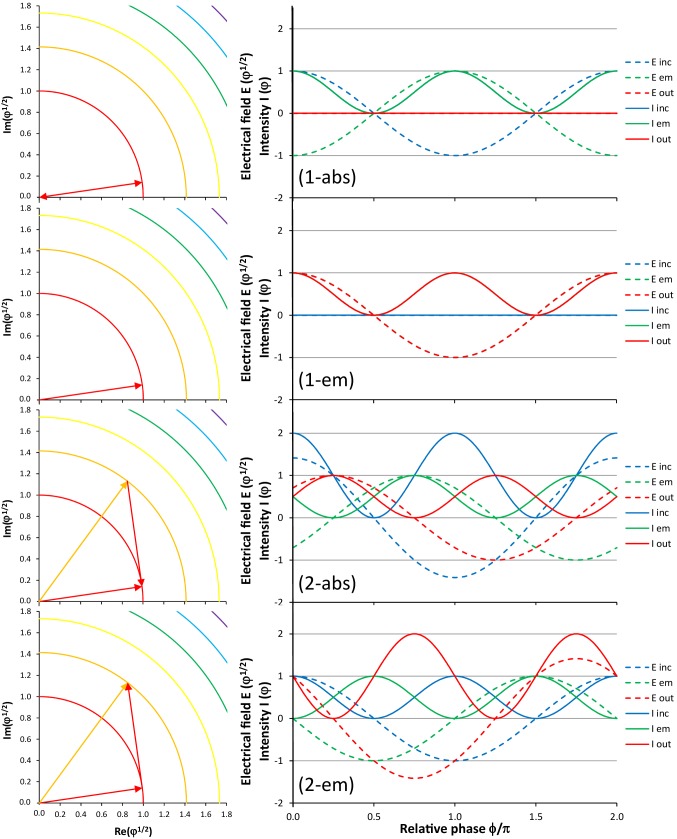
From top to bottom: the four processes of (1-abs) absorption of the electric field of a single incident photon, (1-em) spontaneous emission of the electric field of a photon into an empty mode, (2-abs) absorption of the electric field of one out of two incident photons, and (2-em) stimulated emission of the electric field of a photon driven by a single incident photon. Left column: Individual processes in the amplitude–phase diagram (equivalent to Fig. 2). Right column: electric fields *E* (dashed lines, with the ordinate calibrated as square root of number of photons) and intensities *I* (solid lines, with the ordinate calibrated as number of photons) versus relative phase *ϕ* in units of *π*. Incident (inc, blue) wave, displayed with absolute phase angle zero, as well as emitted (em, green) and resulting (out, red) waves. For (1-abs), blue and green solid lines are identical, and red dashed and solid lines are identical. For (1-em), blue dashed and solid lines, green and red dashed lines, and green and red solid lines, respectively, are identical

These four processes are displayed individually on the left-hand side of Fig. 3. In (1-abs), the rightward-pointing red vector is the incident field, the leftward-pointing red vector is the emitted field, and the resulting field is a vector of length zero at the origin. In (1-em), the incident field is a vector of length zero at the origin, whereas the red vector simultaneously indicates the emitted field and the resulting field. In (2-abs), the orange vector is the incident field, the downward-pointing red vector is the emitted field, and the rightward-pointing red vector is the resulting field. In (2-em), the rightward-pointing red vector is the incident field, the upward-pointing red vector is the emitted field, and the orange vector is the resulting field. The four processes are quantified on the right-hand side as a sum of sine waves by taking into account the different phase angles emerging from the left-hand side; see also [5]. An additional phase angle of 8 degrees has been introduced in the amplitude–phase diagrams to move the arrows away from the *x*-axis for improved visibility. This angle is not considered in the sine-wave diagrams on the right-hand side of Fig. 3. The incident field (blue dashed line) induces the emitted field (green dashed line), thereby generating the resulting field (red dashed line). The corresponding intensities, calibrated as number of photons, are shown as solid lines. In all four examples, the energy is conserved, because the expected number of photons results.

## Inverse processes

The answer to the question raised in the title is subtle. The truly inverse processes are those, in which (abs) an absorbing atom removes one photon from an incident field containing *φ* + 1 photons, such that the resulting field contains *φ* photons, and (em) an emitting atom adds one photon to an incident field containing a number *φ* of photons, such that the resulting field contains *φ* + 1 photons. Both processes comprise the same number of photons and are described by the same vector triangle, with the same phase angles, in the amplitude–phase diagram (Fig. 2). The reader can easily extend the diagram in Fig. 2 to larger numbers of involved photons; see also [5]. This identification of inverse processes is resembled by the results of the Jaynes–Cummings model [9].

In the four cases displayed in Fig. 3, the relative phase angle of emitted field with respect to incident (or resulting) field is, from top to bottom, − *π* (arbitrary), arbitrary (0), − 0.75*π* (− 0.5*π*), and 0.5*π* (0.25*π*), respectively. The common pattern of the two corresponding processes with the same number of photons involved is the following. The relative phase of emitted with respect to resulting field in the absorption process lags by a phase difference of *π* behind the relative phase of emitted with respect to incident field in the emission process, namely − *π* vs. 0, arbitrary vs. arbitrary, − 0.75*π* vs. 0.25*π*, and − 0.5*π* vs. 0.5*π*, respectively.

This leads us to the simple conclusion that the inverse process of (1-abs) absorption of a single incident photon is (1-em) spontaneous emission into an empty mode, whereas the inverse process of (2-abs) absorption of one photon out of an incident field containing two photons is (2-em) stimulated emission of a second photon induced by a single incident photon.

It then also becomes clear that the two processes of (1-abs) absorption and (2-em) stimulated emission induced by one incident photon are not inverse processes. They are neither in phase and anti-phase with the driving field, respectively, nor are they even in opposite phase with each other. In (1-abs) absorption of a single incident photon, the induced atomic oscillation (and the quasi-emitted photon) is in anti-phase with the incident field, thereby extinguishing the incident field. In (2-em) stimulated emission induced by a single photon, the phase of atomic oscillation and emitted photon is 90° in lead of the incident field (Fig. 2).

It is not unlikely that Einstein approached the investigation that led him to the discovery of stimulated emission [2] in the following way. The two-level atoms he considered for the description of blackbody radiation [10] can each only absorb one photon, which they would most likely re-emit by spontaneous emission. Therefore, it is natural to assume one incident photon when describing the process of absorption and no incident photon when describing the process of spontaneous emission, in their simplest form. It is then only consequent to also consider stimulated emission by an excited atom for the simplest case of one incident photon. When comparing these three simplest possible processes, Einstein rightfully identified absorption and spontaneous emission as inverse processes because of their symmetry: one photon either disappears or appears. In contrast, the process of stimulated emission involves two photons. Einstein interpreted stimulated emission as a “new” phenomenon, because it was hitherto unknown and because it does not have a symmetry with the two other processes. Einstein certainly had a point! Unfortunately, although Maxwell’s electromagnetic theory was known, Einstein did not investigate the phase aspect of these processes, which would have revealed the full picture.

## Conclusion

On the one hand, the two processes of absorption and stimulated emission of the electromagnetic field of one photon by an atom driven by the electromagnetic field of a single incident photon are connected by the facts that they are both (1) driven by an external electromagnetic field and (2) quantified by the same Einstein *B* coefficient. However, these two processes are unrelated in terms of the number of photons involved and do not exhibit opposite phase difference between driving and driven field. On the other hand, the two processes of absorption of a single incident photon by an atom and spontaneous emission of one photon from an atom into an empty mode are connected by the facts that (3) they are inverse processes in the amplitude–phase diagram, with the same phase angles, and (4) the same number of photons is involved. The truly inverse process of stimulated emission of one photon by an excited atom driven by a single incident photon is the absorption of one out of two incident photons by an atom in its ground state, as both processes (1) are driven by an external electromagnetic field, (2) are quantified by the same Einstein *B* coefficient, (3) are opposite processes in the amplitude–phase diagram, with the same phase angles, and (4) involve the same number of photons. Hence, neither Einstein nor Lamb was right or wrong.
